# Risk Factors for Hospitalisation amongst Leptospirosis Patients in New Zealand

**DOI:** 10.3390/tropicalmed6040188

**Published:** 2021-10-20

**Authors:** Maryna Sokolova, Jonathan C. Marshall, Jackie Benschop

**Affiliations:** 1 EpiCentre, School of Veterinary Science, Massey University, Palmerston North 4474, New Zealand; 2 School of Fundamental Sciences, Massey University, Palmerston North 4474, New Zealand; j.c.marshall@massey.ac.nz; 3 Molecular Epidemiology and Public Health Laboratory, Hopkirk Research Institute, School of Veterinary Science, Massey University, Palmerston North 4474, New Zealand; j.benschop@massey.ac.nz

**Keywords:** hospitalisation, risk factors, surveillance data, *Leptospira* spp.

## Abstract

Leptospirosis is a neglected zoonotic disease that is widespread in tropical and subtropical regions such as Oceania, which includes New Zealand. The incidence rate of leptospirosis in New Zealand remains high in comparison to other high-income countries, with over half of the notified patients hospitalised, and the factors associated with hospitalisation are poorly understood. This study aimed to estimate the risk factors for hospitalisation amongst leptospirosis patients using passive surveillance data: notifications from 1 January 1999 to 31 December 2017 extracted from New Zealand’s notifiable disease database. There were 771 hospitalised and 673 non-hospitalised patients. Multivariable logistic regression was used to identify risk factors. The year of notification was significantly and positively associated with hospitalisation, with adjusted (adj.) OR 1.03 (95% CI:1.01–1.05). Occupation was significantly associated with hospitalisation, with the adjusted odds of hospitalisation amongst dairy farmers notified with leptospirosis at adj. OR 1.44 (95% CI: 1.02–2.02) times the adjusted odds of hospitalisation amongst farmers that worked with other livestock. Seropositivity for *Leptospira interrogans* Copenhageni (adj. OR 5.96, 95% CI: 1.68–21.17) and Pomona (adj. OR 1.14, 95% CI: 0.74–1.74)) was more likely to result in hospitalisation when compared to *Leptospira borgpetersenii* Ballum (baseline). Seropositivity for *Leptospira borgpetersenii* Hardjo (adj. OR 0.71, 95% CI: 0.49–1.01) and Tarassovi (adj. OR 0.39, 95% CI: 0.23–0.66) was less likely to result in hospitalisation when compared to Ballum (baseline). All the estimates were additionally adjusted for the effect of sex, age, ethnicity, reported occupational exposure, geographical location, reported season, and deprivation status Although passive surveillance data has limitations we have been able to identify that the New Zealand dairy farming population may benefit from a targeted awareness campaign.

## 1. Introduction

Leptospirosis, caused by pathogenic members of the *Leptospira* genus, is a neglected, re-emerging zoonotic disease that is distributed worldwide. An assessment of the burden of leptospirosis by the World Health Organization (WHO) published in 2011 estimated the global annual incidence of endemic human leptospirosis as exceeding five severe cases per 100,000 people, excluding cases from outbreaks [1]. A systematic review of the literature on leptospirosis morbidity and mortality from January 1970 to October 2008 estimated 1.03 million leptospirosis cases annually, resulting in 58,900 deaths [2].

In New Zealand leptospirosis is predominantly a disease of farm workers and meat workers [3], and four serovars *Leptospira borgpetersenii* Hardjo, Ballum and Tarassovi, and *Leptospira interrogans* Pomona predominate amongst the notifications when a serovar is identified [4]. The introduction of livestock leptospirosis vaccination programmes in NZ in the 1970s was associated with a significant drop in the incidence rate in human leptospirosis from 30/100,000 people in the 1970s [5] to 4.4/100,000 in the 1990s [6], and the average annual incidence from 1999–2017 was 2.01/100,000 [7]. The long-term decrease in incidence was primarily due to the reduction in the Pomona and Hardjo cases, while the incidence attributed to the non-vaccine serovars, Ballum and Tarassovi, increased [6]. Currently, dairy cattle-associated Pomona, Hardjo, and *L. interrogans* Copenhageni and pig-associated Pomona and Tarassovi are largely controlled via vaccination programmes, with an estimated 99.5% of the dairy herds [8] and commercial swine farms [9] complying. However, vaccination rates in dry stock farming are low, with approximately 20% of beef herds, 10% of deer herds, and less than 1% of sheep flocks vaccinated [10].

Humans are infected with *Leptospira* via direct contact with an infected animal’s urine or indirectly via contaminated water or other environmental vectors. The incubation period ranges from 2–30 days, typically averaging 10 days [11]. Leptospirosis often presents with non-specific febrile symptoms, such as headache and myalgia, which may progress to hepatorenal failure and death [11]. Most cases are self-limiting and, likely, undiagnosed [12]. In New Zealand (NZ), patients usually present to general medical practitioners [13,14] or, if the disease is more severe, to hospital Emergency Departments (EDs) [15], where patients may be admitted to a general ward or intensive care/high dependency unit. If the latter, symptoms may include myalgia, headache, nausea, and vomiting [15]. Death is a rare outcome in NZ [10].

The laboratory tests normally performed in New Zealand are serology tests, polymerase chain reaction (PCR) assays, and culture [16]. MAT is the standard serology test used for confirmation of leptospirosis [16], while the other serology test, IgM Elisa, is mostly used for screening [16]. Both PCR and culture are also used as confirmatory tests.

Leptospirosis is still one of the most important occupationally acquired zoonotic diseases in New Zealand. Its average 19-year incidence is estimated at 2.0/100,000 in the general population and 2.24/100,000 in the Māori population [7]. Hospitalisations among notified cases appear to be increasing, with approximately 50% of cases hospitalised, and, according to the Institute of Environmental Science and Research (ESR) report from 2019 [4], 63% of cases reported in 2017 were hospitalised. Hospitalisation costs form a significant part of the healthcare burden [17], and hospitalisations are likely to reflect the severity of the disease. With two out of every three patients being hospitalised and a median length of stay of 6 days [15], there is a need to identify factors associated with hospitalisation to inform and appropriately address public health messaging to reduce hospitalisations. The specific objective of this study was to identify the risk factors for hospitalisation in patients with leptospirosis in New Zealand.

## 2. Materials and Methods

### 2.1. Data Sources

Routinely collected surveillance data of 1627 leptospirosis notifications from 1 January 1999 to 31 December 2017 was extracted from NZ’s notifiable disease database. In NZ, human leptospirosis is a mandatory notifiable disease [18]. Briefly, the leptospirosis notification process [18] starts with medical practitioners or other health professionals reporting suspected leptospirosis cases to the medical officers of health, who then interview confirmed and probable leptospirosis cases in order to record surveillance data in the standard case report forms [19]. A ‘Confirmed’ case of leptospirosis had a clinically compatible illness and one or more positive laboratory test results, such as (i) a four-fold increase between paired sera by two consecutive tests conducted in the same laboratory in the MAT; (ii) a single MAT with a serological titre of ≥400; (iii) a nucleic acid detection in clinical specimen (iv) a positive culture. A ‘Probable’ case was defined by a clinically compatible illness and a single MAT test showing a titre of <400 [18]. The case report form information is linked with the available serovar data from the *Leptospira* Reference Laboratory and entered into NZ’s notifiable disease database. Thus, the available patient data include age, sex, hospitalisation, and diagnostic status, in addition to the information outlined below.

The serovar was recorded as reported by testing laboratories. From all notified cases, the MAT result was recorded for 1178/1627 cases and culture test result was recorded for 311/1627 cases. In New Zealand, eight serovars are included in the MAT testing panel, which includes the following serovars: Ballum, Copenhageni, Hardjo, Pomona, Tarassovi and *Leptospira* interrogans serovars Australis, Canicola, Grippotyphosa, *Leptospira* interrogans Serovar Bratislava was included in the MAT panel until 2009. Hence, the surveillance data on this serovar were also available until 2009.

The self-reported ethnicity is recorded as per the NZ convention of prioritised ethnicity [19]. The patients’ deprivation index (NZDep) is assigned based on their home address. NZDep [20] is a ranking system of socio-economic population attributes such as income, education, and employment that is assigned to small areas or meshblocks defined by Statistics New Zealand (StatsNZ), with a population of around 60–110 people. The patient’s rurality status classification follows existing NZ urban and rural boundaries with seven categories [21]. Occupations are recorded as per the Australian and NZ standard classifications of occupations [22]; however, there is an additional and distinct occupation-related variable in the database called reported occupational exposure (ROE). The distinction is that occupation is a patient’s usual work, while ROE is the patient’s reported exposure to *Leptospira* during the incubation period of 4–20 days while performing work-related activities. The database also contains overseas travel history and animal and water exposure, recorded as “yes” if the patient reported travelling, had contact with wild or domestic animals, or participated in any freshwater activity in the 4–20 days before illness, otherwise these data are recorded as “no” or “unknown”.

Cases are attributed to their District Health Board (DHB) locality, as in NZ public health and disability services are governed and distributed by DHBs, of which there are 20, each operating within their designated geographical area. In addition, the database contains the year and date of notification.

### 2.2. Data Categorisation

#### 2.2.1. Diagnostic Status, Travel, and Hospitalisation

All cases with a diagnostic status recorded as ‘Probable’ or ‘Confirmed’ were retained for analysis; those ‘Under Investigation’ and ‘Not a Case’ were excluded. Only locally acquired cases were included in the analysis; thus, patients who had reported travelling overseas during the incubation period were excluded. The remaining cases were then filtered by their hospitalisation status, and only cases with known hospitalisation status were retained. Thus, hospitalisation status (the outcome variable) had two levels defined: ‘Yes’ and ‘No’.

#### 2.2.2. Demographic Categories

For the patient age categorisation, we used natural tercile age breaks: ‘Young’, from 0–36 years; ‘Middle age’, from 37–49 years; and ‘Senior’, from 50 years and older. From the total of 1444 cases in this dataset, there were two notified cases for small children (<5 years) and one notified case in the young children group (5–16); therefore, due to the small sample size, the children were categorised into the ‘Young’ age group. Sex was defined as either male or female. NZ is a multicultural country, with Europeans being the largest ethnic group, comprising 70.2% of the population, followed by Māori at 14.6%, and Pacific people at 8.6%, as reported by the 2018 Census. For our analysis, we categorised ethnicities into three categories: ‘Māori and Pacific Peoples’; ‘European and Other’; and ‘Unknown’, where the ethnicity code was not assigned, or the record was blank. The deprivation Index levels 1, 2, and 3 were categorised as ‘Least Deprived’; levels 4, 5, and 6 as ‘Moderately Deprived’; and levels 7–10 as ‘Most Deprived’; any missing data were classified as ‘Unknown’. Eighty-four percent of New Zealanders live in urban areas. Urban/rural profiles were re-classified into two main categories, ‘Rural’ and ‘Urban’, or where the profile was not recorded, ‘Unknown’.

#### 2.2.3. Putative Risk Factors

Seven separate serovar categories were created. The endemic *Leptospira borgpetersenii* serovars Ballum, Hardjo, and Tarassovi and the *Leptospira interrogans* serovars Copenhageni, and Pomona formed five categories. The non-endemic *Leptospira interrogans* serovars Australis, Canicola, Grippotyphosa, and Bratislava were merged into an ‘Exotic’ category. An ‘Unknown’ group, which included all cases where the serovar was not identified for any reason, such as missing data, blank entry, or non-identified serovar entries, was the seventh category.

As leptospirosis in NZ is an occupational hazard for those who are working with farm animals, we focused analysis on farm animal-related occupational groups. Occupations were divided into the following four categories: ‘Dairy Farmer’, ‘Non-Dairy Farmer’, ‘Meat Worker’, and ‘Other’—the latter including all occupations that did not fit into the first three categories, such as unemployed, missing data, and undefined occupation. The ‘Dairy Farmer’ category included all occupations self-identified by the inclusion of the word ‘dairy’ or ‘milker’, such as sharemilker, relief milker, dairy farm worker, and others. The ROE was categorised as ‘Yes’ exposed, ‘No’ not exposed, and ‘Unknown’ when not known by the patient. Animal exposure was categorised as ‘Yes’ exposed or ‘No’ not exposed. Water exposure was categorised as ‘Yes’ or ‘No’.

#### 2.2.4. Spatial and Temporal Categories

We re-organised the reported cases from their corresponding geographical location by DHB to the three corresponding geographical areas of South Island (SI), Upper North Island (UNI), and Lower North Island (LNI). The southern and the eastern boundaries of Waikato DHB [23] defined a cut point line between LNI and UNI. The report year (year of notification) was left as a continuous variable, and the months when the cases were notified were categorised into four groups by the southern hemisphere seasons: summer (December–February), autumn (March–May), winter (June–August), and spring (September–November).

### 2.3. Statistical Methods

#### 2.3.1. Descriptive Data Analysis

Descriptive data analyses included contingency tables, proportion tests, χ^2^ tests, boxplots, and bar plots to explore the relationship between hospitalisation and the putative risk factors. The Goodman-Kruskal asymmetric test [24] was applied to compute both the forward and the backward associations between all the pairwise combinations of categorical variables in the data frame to assess collinearity. Multi-collinearity was considered present if the coefficients exceeded 0.8.

#### 2.3.2. Logistic Regression Model Building

Bivariate associations between hospitalisation and the putative risk factors were explored by logistic regression. The criterion for the inclusion of variables into the preliminary final model was *p* ≤ 0.2, obtained in the bivariate logistic model.

A multivariable logistic regression model was built by forward stepwise selection, followed by backward elimination based on a Likelihood Ratio Test (LRT, significance level *p* ≤ 0.05) to confirm the inclusion/exclusion of variables into/from the preliminary final model.

Confounding variables were identified by a change of the coefficient of any other variable of more than 20% in the presence of the confounder and by the confounding variables’ association with both the dependent and the independent variables, assessed by χ^2^ tests, as recommended by Dohoo et al. [25]. In cases when any other predictor was found to be a confounder, it was retained in the model regardless of its statistical significance.

Null, preliminary, and final models were compared by ANOVA, LRT, and Akaike Information Criterion (AIC) [26]. The fit of the model to the data was established by the Goodness of Fit test [27]. The performance of the model was evaluated by the measures of the LRT and AIC. The ROC test established the predictive ability of the model. The best-performing final model was selected on the combined basis of the following test results: (i) Highest value of AUROC (Area Under Receiver Operating Curve); (ii) lowest value of AIC; (iii) significance of LRT. The fit of the data to the model was evaluated by assessing outliers using Pearson residuals and by evaluating influential data points using Cook’s distance.

All data analysis was performed in RStudio software [28] with the packages epiR [29], car [30], tidyr [31], ResourceSelection [32], ROCR [33], ggplot2 [34], sjPlot [35], GoodmanKruskal [36], lmtest [37], and RAWGraphs [38]. 

This was a minimal risk study involving a retrospective analysis of de-identified data and was recorded on the Massey University Low Risk database (Reference: 4000020417). Cultural consultation occurred with a senior Māori researcher.

## 3. Results

### 3.1. Descriptive Analysis

**Figure 1:**
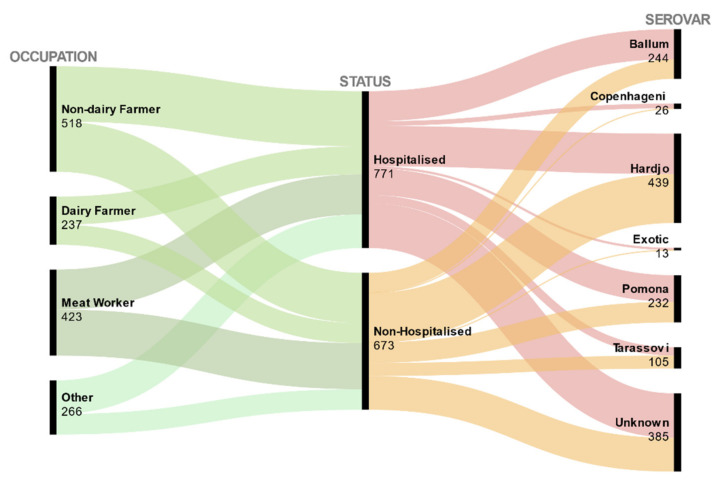
Alluvial diagram of counts of Occupation and Hospitalisation categories amongst notified cases of leptospirosis in New Zealand 1999–2017 by Serovar and Occupation categories represented as flows. Each black rectangle represents a category, its height proportional to its size. Correlations between categories are shown by curved ribbons whose width is proportional to group and correlation size.

**Figure 2:**
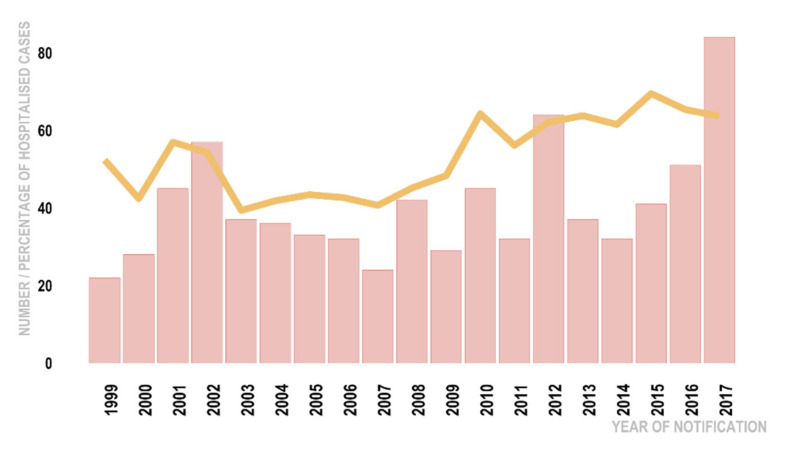
Hospitalised notified leptospirosis cases by year, New Zealand 1999–2017. Pink bars represent the number of hospitalised cases, and the yellow line represents percentage of hospitalised cases.

The descriptive characteristics of the variables are presented in Figure 1 and Table 1, Table 2 and Table 3. Figure 1 shows the number of reported cases in the Occupational and Serovar categories, organised by their hospitalisation status. From 1627 cases notified during the 1999–2017 period, 1444 (89%) cases met the inclusion criteria when filtered by their diagnostic and travel statuses and hospitalisation. Of these, MAT result was available for 73% (95% CI 71–76%) of cases and culture results were recorded for 19% (95% CI 17–21%) of cases; 53% (95% CI: 51–56%) or 771/1444 patients were hospitalised (Figure 1), The percentages of hospitalisation varied within different categories. For example, by ethnicity, in the Māori and Pacific People category more than half, 57% (95% CI: 50–63%), were hospitalised whilst in the Europeans and Others group less than half, 45% (95% CI 41–57%), were hospitalised. The percentages of hospitalisation by sex were similar at 53% (95% CI: 51–56%) for males and 55% (95% CI: 46–53%) for females. There was a general increase over time in the number of hospitalisations: the number of hospitalised cases fluctuated from as low as 22 in 1999 to a maximum of 84 in 2017 (Figure 2), with an average of 41 (95% CI: 33–48%) hospitalisations per year. No statistically significant correlation was detected between the 15 explanatory variables of interest selected for bivariate analysis from the dataset, as none of the Goodman-Kruskal coefficients exceeded 0.3. The bivariate associations between the hospitalisation and demographic variables, the putative risk factors, and the spatial and temporal variables are presented in Table 1, Table 2 and Table 3. Notwithstanding our removal from the dataset of cases who had travelled overseas during the incubation period (n = 72), 57% (13/23) of the notified cases of leptospirosis caused by the Exotic group of serovars (Table 2) remained in the dataset.

### 3.2. Multivariable Model

The final multivariable logistic regression model included the following variables: hospitalisation, report year, occupation, serovar, geographical location, season, and deprivation status. Age, ROE, and ethnicity were included as confounders.

Seropositivity for *Leptospira interrogans* Copenhageni (adj. OR 5.96, 95% CI: 1.68–21.17), the Exotic serovar group (adj. OR 3.37, 95% CI: 0.71–15.97), and Pomona (adj. OR 1.14, 95% CI: 0.74–1.74)) was more likely to result in hospitalisation when compared to *Leptospira borgpetersenii* Ballum (baseline). Seropositivity for *Leptospira borgpetersenii* Hardjo (adj. OR 0.71, 95% CI: 0.49–1.01) and Tarassovi (adj. OR 0.39, 95% CI: 0.23–0.66)) was less likely to result in hospitalisation when compared to Ballum (baseline) (Table 4). The adjusted odds of hospitalisation for dairy farmers were 1.44 (95% CI: 1.02–2.02) times the odds of hospitalisation for non-dairy farmers. The year of notification was significantly and positively associated with hospitalisation, with adj. OR 1.03 (95% CI: 1.01–1.05). Although not statistically significant at *p* = 0.06, the adjusted odds of hospitalisation for leptospirosis patients who identified as Māori and Pacific People were 1.39 (95% CI: 0.99–1.96) times the odds of hospitalisation for leptospirosis patients from the European and Other ethnicities. Deprivation status was significantly associated with hospitalisation; patients from the ‘least deprived’ category were more likely to be hospitalised, compared to patients from the ‘most deprived’ category (adj. OR 1.64, 95% CI: 1.15–2.33). Leptospirosis patients from the South Island were half as likely to be hospitalised (adj. OR 0.53, 95% CI: 0.31–0.91) when compared to patients from the Lower North Island.

## 4. Discussion

This is the first study of the risk factors for hospitalisation amongst notified leptospirosis patients in NZ. We have identified that with infection with *Leptospira interrogans*, the dairy farming occupation and Māori or Pacific ethnicity place leptospirosis patients at increased risk of hospitalisation. Leptospirosis patients seropositive to serovars within the L. interrogans species were more likely to be hospitalised than patients seropositive to *Leptospira borgpetersenii*, suggesting that the former species has greater pathogenicity. In particular, we found that patients seropositive to *Leptospira interrogans* Copenhageni (adj. OR 5.96, 95% CI: 1.68–21.17), the Exotic serovar group (adj. OR 3.37, 95% CI: 0.71–15.97), or Pomona (adj. OR 1.14, 95% CI: 0.74–1.74) were more likely to be hospitalised when compared to the patients seropositive to *Leptospira borgpetersenii* (Ballum (baseline), while patients seropositive to Hardjo (adj. OR 0.71, 95% CI: 0.49–1.01) and Tarassovi (adj. OR 0.39, 95% CI: 0.23–0.66) were less likely to be hospitalised when compared to the patients seropositive to *Leptospira borgpetersenii* (Ballum (baseline)). This finding is also supported by overseas research [39], where the risk of severe leptospirosis in hospitalised patients in New Caledonia was higher (OR = 2.79, 95% CI 1.26–6.18) in the patients seropositive to *L. interrogans* when compared to the patients seropositive to other *Leptospira* species. *L. interrogans* interacts directly with human platelets in vitro and this has been postulated as causing the platelet dysfunction associated with haemorrhagic diathesis, frequently observed in severe cases of leptospirosis [40]. Thus, different serovars display various pathogenicity [40], and, according to our results, the serovars classified as Exotic, along with Copenhageni and Pomona, may result in more severe disease. The latter two serovars’ cytotoxic ability [41] is likely to cause increased disease severity.

Our finding of the serovar-associated risk for hospitalisation supports the importance of livestock vaccination against leptospirosis, particularly vaccination against the serovars Copenhageni and Pomona, for both dairy and dry stock. In NZ, the dry stock are pasture-grazed beef cattle, sheep, and deer farmed for meat, wool, and velvet production [42]. While the ongoing vaccination of all dairy herds [8] and commercial pig herds remains crucial, efficacious vaccines [43,44] are available for other classes of dry stock in NZ, such as sheep and beef cattle, but their uptake in these classes of stock is limited [45]. The vaccination of all classes of livestock is an important preventative measure against severe human cases of leptospirosis; however, the choice of available vaccine should also reflect on the most commonly identified serovars in both humans and livestock. For instance, the most reported serovars in the human notification data in New Zealand from 1999–2017 were Hardjo at 41.8% (492/1178), Pomona at 22.4% (264/1178), Ballum at 21.4% (253/1178) and Tarassovi at 9.3% (109/1178) [7]. Livestock vaccines cover Hardjo, Pomona, and Copenhageni. Specific seroprevalences have also been reported for each class of livestock [8,10,43,46], and showed that seroprevalence by serovar varied within different classes of livestock, but serovar Pomona [47] and serovar Hardjo were highly prevalent. While *L. interrogans* serovar Copenhageni is not highly prevalent in NZ livestock [48] or human notifications [7], the high risk of hospitalisation due to Copenhageni (adj. OR 5.96, 95% CI: 1.68–21.17) is critical. Rats are recognised as a source of infection of Copenhageni for humans and other animals [49,50]; therefore, rodent control is also an important cornerstone of leptospirosis prevention.

Our results indicate that the risk of hospitalisation may be increasing annually; thus, the severity of leptospirosis in NZ over the study period (1999–2017) may also have increased. However, the increase in hospitalisations may also be due to the patients’ health-seeking behaviour and/or clinicians’ diagnostic suspicion having reduced over the study period. The reduction of clinical suspicion or health-seeking behaviour may be associated with increased hospitalisation because late diagnosis or patient presentation may lead to the increased severity of leptospirosis, which may require hospitalisation. An Australian study by Lau et al. [51] highlighted the importance of the clinicians’ diagnostic suspicion of patients within high-risk groups and sought to improve awareness of leptospirosis among GPs. McLean et al. [14] and Earl et al. [16] highlighted the importance of high clinical suspicion of leptospirosis among GPs and the choice [52] and timing of diagnostic tests for leptospirosis in NZ.

Occupation was significantly associated with hospitalisation amongst leptospirosis patients. Dairy farmers with leptospirosis were 44% (adj. OR, 1.44 (95% CI: 1.02–2.02) more likely to be hospitalised than farmers with leptospirosis who worked with non-dairy stock (Table 4). This association may relate to health-seeking behaviours or contributing factors, e.g., reluctance to seek help while working alone, continuing to work when ill, social isolation, long working hours, financial pressure [53], or the farm’s remote location. A combination of these behaviours and factors may lead to a health-seeking delay in the dairy farmer group and may result in increased disease severity and hospitalisation because of the delayed patient presentation, clinical suspicion, and treatment. For example, the incidence rate of GP visits by NZ dairy farmers in 2005–2014 was noticeably lower than that of the sheep and beef farmers [54]. These figures are not adjusted for age; however, they support the health-seeking hypothesis.

Another possible explanation for the higher odds of hospitalisation in dairy farmers is that they are exposed to a higher bacterial load at work than those working in other animal-contact occupations. Non-dairy animal-contact occupations are likely to have much less frequent contact with animal urine. Dairy farmers and dairy workers are exposed to cattle urine twice a day when milking, as cows stand on the milking platform above the worker’s height in both the herringbone and the rotary dairy shed types. In addition, the indirect exposure of dairy farmers to cows’ urine occurs during the twice-a-day post-milking cleaning of surfaces and equipment with water and during spray fertilisation of the pasture with effluent. Findings of a study that measured dose-response models using golden hamsters under experimental conditions [55] support the hypothesis of higher bacterial load leading to more severe disease. Specifically, the golden hamsters (*Cricetus auratus*) were inoculated with a range of pathogenic *Leptospira* doses through various routes and the results showed that the higher bacterial load led to more severe leptospirosis. In the absence of human dose-response models, the hamsters’ serological and clinical responses to leptospirosis are the closest to those of humans [46], suggesting hamsters as a reasonable model for human infection. Therefore, the higher odds of severe leptospirosis, as measured by hospitalisation, in dairy farmers may be partially attributed to a high frequency of direct or indirect contact with cattle urine and consequently to a potentially higher bacterial load. In further support, a meta-analysis of 14 articles reporting the quantities of *Leptospira* shed in the urine of various mammals estimated that cattle release the highest concentration, adjusted for the large urine volume, of *Leptospira* at 6.3 × 10^8^ bacteria per ml of urine per day compared to other species, thus identifying cattle as an important source of *Leptospira* [56]. Although 99.5 % of dairy herds in NZ are vaccinated [8], 27% (53/199) of vaccinated dairy herds show evidence of shedding *Leptospira* [8]; therefore, vaccinated dairy herds may be a source of infection for dairy farmers.

An outreach programme explaining the flu-like symptoms of leptospirosis and the importance of early intervention to dairy farmers could help to reduce the severity of the disease amongst notified cases of dairy farmers through their early presentation, their GP’s suspicion, and antibiotic administration. The beneficial effects of early antibiotic treatment on the severity of the disease were noted by Haake and Levett [12], citing findings from Barbados [57] and the Philippines [58] of placebo-controlled studies using intravenous penicillin in the treatment of leptospirosis. In NZ, antibiotics are commonly prescribed to patients for the treatment of leptospirosis during their initial visit to primary medical care before laboratory confirmation. This was determined by two case series of leptospirosis, where 87% (41/47) [16] and 100% (11/11) [59] of patients suspected of leptospirosis were prescribed antibiotics during their initial visit to primary care.

Our results indicate that the risk of hospitalisation may be higher for Māori and Pacific people than for NZ European and Other. The ethnic disparity in hospitalisation may be due to differential access to healthcare or inappropriate health messaging. Regional Māori Relationship Boards are working with us to identify people and groups within Māori communities to support the dissemination of information on leptospirosis.

In this discussion, we used hospitalisation to approximate disease severity. However, it is important to note that hospitalisation may not accurately indicate the disease severity. For example, the length of hospital stays may vary, e.g., from overnight to many nights, and the nature of the hospitalisation may vary, e.g., ward vs. ICU admission, and variability in the severity occurs amongst hospitalised patients. For example, a dairy-farm outbreak investigation of three leptospirosis patients in 2015 reported two patients having overnight stays in a ward and one patient staying 7 days and being admitted to ICU [13]. Furthermore, in rural NZ, where many leptospirosis patients are diagnosed, there are small regional hospitals where clinicians with a lower threshold for admission than clinicians in city hospitals may admit patients overnight for observation. This will often be due to the remoteness of the patient’s place of residence [60]. Nevertheless, in this passively collected surveillance dataset, hospitalisation was the only factor available to us that could be used to approximate severity. Other studies have used a similar approach to investigate risk factors for severe disease in passive surveillance data sets [61,62].

The presence of cases caused by the Exotic serovars in this study was an unexpected finding, as all cases attributed to the Exotic serovars were expected to be linked to the overseas-acquired status in the ESR dataset; if a patient returned to NZ 4–20 days prior to the illness onset date, this case was identified by ESR as overseas-acquired. It is generally accepted that the leptospirosis incubation period can be as long as 30 days [41]. However, in this study, when travel history was used in conjunction with the incubation period of 4–20 days to separate an overseas-acquired case from local cases, 57% (13/23) of cases by the Exotic group of serovars remained in the dataset and, therefore, were identified as locally acquired. The presence of notified locally-acquired cases by Exotic serovars in this analysis could be attributed to the application of an incubation period of leptospirosis of 4–20 days to a travel history of the patient by ESR; it is possible that the application of a more extended incubation period could have reduced the number of locally-acquired cases by the Exotic group of serovars in this analysis. However, other factors such as a poor recall of travel by patients when interviewed by a medical officer or locally-acquired leptospirosis with no travel history outside 30 days prior to diagnosis caused by the Exotic serovar, or cross-reactivity of the MAT to other serovars in the individual serogroup [41] could also explain this finding.

One of this study’s limitations is the use of a MAT test as a serovar identifier. While the MAT is the reference test recommended by the WHO and the International Committee on Systematic Bacteriology and the Subcommittee on the Taxonomy of *Leptospira*, it is considered serogroup rather than serovar specific [63]. However, where a limited number of serovars are known to be present, serovars can be identified by MAT with a high degree of certainty [18]. Although identification of the infecting serovar by MAT is limiting, our study contains surveillance data on the notified cases of 1999–2017, and the MAT test result was the most comprehensive serovar information available for use at the time.

We partially controlled confounding by the inclusion of all identified confounders into the multivariable logistic model. However, the potential for unmeasured confounding to occur in the analyses of routinely collected surveillance data is strong as limited data are available. In future, the data quality could be enhanced by adding information related to disease severity, such as hospital discharge data, which could be sourced from The National Minimum Dataset (NHMDS) collection, publicly available from the MOH. Furthermore, data collected with a bespoke questionnaire would be a more appropriate alternative to surveillance data when specific research questions need to be addressed.

The current study’s findings may be used to inform and target public health messaging by reaching out to dry stock farming industry bodies and stakeholders to improve the livestock vaccination programme uptake across stock classes; to dairy farmers and workers to explain flu-like symptoms of leptospirosis and the importance of early intervention; and to GPs to raise the suspicion of leptospirosis.

## Figures and Tables

**Table 1:** Demographic variables and crude odds ratios for hospitalisation amongst notified cases of leptospirosis in New Zealand 1999–2017.

	Hospitalised (N = 771)		Non-Hospitalised (N ^1^ = 673)		Hospitalisation Rate	Bivariate Analysis Results		
Demographic Variables	Number (n)	% (n/N)	Number (n ^1^)	% (n ^1^/N ^1^)	n/(n + n ^1^)	β-Coefficient	Crude OR (95% CI)	*p*
Age								
Young (0–36)	255	33	237	35	0.52	Baseline		
Middle Age (37–49)	247	32	238	35	0.51	−0.04	0.96 (0.75, 1.24)	0.78
Senior (>50)	269	35	198	29	0.58	0.23	1.26 (0.98, 1.62)	0.07
Sex								
Female	75	10	62	9	0.547	Baseline		
Male	696	90	611	91	0.533	−0.06	0.94 (0.66, 1.34)	0.73
Ethnicity								
European and Other	590	77	509	76	0.54	Baseline		
Māori and Pacific Peoples	145	19	111	16	0.57	0.12	1.13 (0.85, 1.48)	0.39
Unknown	36	5	53	8	0.40	−0.53	0.59 (0.38, 0.91)	0.02 *
Deprivation Status								
Most Deprived	241	31	233	35	0.51	Baseline		
Least Deprived	167	22	105	16	0.61	0.43	1.54 (1.14, 2.08)	0.01 *
Moderately Deprived	222	29	174	26	0.56	0.21	1.23 (0.94, 1.62)	0.12
Unknown	141	18	161	24	0.47	−0.17	0.85 (0.63, 1.13)	0.26
Rurality								
Rural	396	51	322	48	0.55	Baseline		
Unknown	68	9	68	10	0.50	−0.29	0.75 (0.52, 1.07)	0.11
Urban	307	40	277	41	0.53	−0.1	0.90 (0.72, 1.12)	0.35

OR = Odds Ratios, *p* = *p*-value, CI = Confidence Interval, * = Statistically Significant, N = Number of Hospitalised Cases, n = Number of Hospitalised Cases in Each Category, N ^1^ = Number of Non- Hospitalised Cases, n ^1^ = Number of Non- Hospitalised Cases in Each Category.

**Table 2:** Putative risk factors and crude odds ratios for hospitalisation amongst notified cases of leptospirosis in New Zealand 1999–2017.

	Hospitalised (N = 771)		Non-Hospitalised (N = 673 ^1^)		Hospitalisation Rate	Bivariate Analysis Results		
Putative Risk Factors	Number (n)	% (n/N)	Number (n ^1^)	% (n ^1^/N ^1^)	n/(n + n ^1^)	β-Coefficient	Crude OR (95% CI)	*p*
Serovar								
Ballum	148	19	96	14	0.61	Baseline		
Exotic	11	1	2	0.3	0.85	1.27	3.57 (0.77, 16.44)	0.1
Copenhageni	23	3	3	0.4	0.88	1.6	4.98 (1.45, 17.02)	0.01 *
Hardjo	198	26	241	36	0.45	−0.63	0.53 (0.39, 0.73)	<0.01 *
Pomona	131	17	101	15	0.56	−0.17	0.84 (0.58, 1.21)	0.35
Tarassovi	41	5	64	10	0.39	−0.87	0.42 (0.26, 0.66)	<0.01 *
Unknown	219	28	166	25	0.57	−0.16	0.85 (0.61, 1.19)	0.35
Occupation								
Non-Dairy Farmer	271	35	247	37	0.52	Baseline		
Dairy Farmer	138	18	99	14	0.58	0.24	1.27 (0.93, 1.73)	0.13
Meat worker	197	25	226	34	0.47	−0.23	0.79 (0.61, 1.02)	0.08
Other	165	21	101	15	0.62	0.4	1.49 (1.10, 2.01)	<0.01 *
ROE								
No	126	16	74	11	0.63	Baseline		
Unknown	89	12	73	11	0.55	−0.33	0.72 (0.47, 1.09)	0.12
Yes	556	72	526	78	0.51	−0.48	0.62 (0.45, 0.85)	<0.01 *
Exposure to Animals								
No	40	5	30	4	0.57	Baseline		
Unknown	51	7	36	5	0.59	0.06	1.06 (0.56, 2.01)	0.85
Yes	680	88	607	90	0.53	−0.17	0.84 (0.52, 1.37)	0.48
Water Exposure								
No	616	80	535	79	0.54	Baseline		
Unknown	155	20	138	21	0.53	−0.02	0.98 (0.75, 1.26)	0.85

OR = Odds Ratios, *p* = *p*-value, CI = Confidence Interval, * = Statistically Significant, N = Number of Hospitalised Cases, n = Number of Hospitalised Cases in Each Category, N ^1^ = Number of Non- Hospitalised Cases, n ^1^ = Number of Non- Hospitalised Cases in Each Category.

**Table 3:** Spatial and crude odds ratios for hospitalisation amongst notified cases of leptospirosis in New Zealand 1999–2017.

	Hospitalised (N = 771)		Non-Hospitalised (N = 673 ^1^)		Hospitalisation Rate	Bivariate Analysis Results		
Spatial and Temporal Variables	Number (n)	% (n/N)	Number (n ^1^)	% (n ^1^/N ^1^)	n/(n + n ^1^)	β-Coefficient	Crude OR (95% CI)	*p*
Report Year						0.06	1.06 (1.03, 1.08)	<0.01 *
Season								
Autumn	215	28	174	26	0.55	Baseline		
Spring	160	21	196	29	0.45	−0.41	0.66 (0.49, 0.88)	<0.01 *
Summer	203	26	152	23	0.57	0.08	1.08 (0.81, 1.44)	0.6
Winter	193	25	151	22	0.56	0.03	1.03 (0.77, 1.38)	0.82
Geographical Location								
Lower North Island	321	42	284	42	0.53	Baseline		
South Island	162	21	189	28	0.46	−0.28	0.76 (0.58, 0.99)	0.04 *
Upper North Island	288	37	200	30	0.59	0.24	1.27 (1.00, 1.62)	0.05 *

OR = Odds Ratios, *p* = *p*-value, CI = Confidence Interval, * = Statistically Significant, N = Number of Hospitalised Cases, n = Number of Hospitalised Cases in Each Category, N ^1^ = Number of Non- Hospitalised Cases, n ^1^ = Number of Non- Hospitalised Cases in Each Category.

**Table 4:** Results from a multivariable logistic regression model of risk factors associated with hospitalisation amongst notified leptospirosis cases in New Zealand 1999–2017.

Variable	β-Coefficient	SE	Adj. OR (95% CI)	*p*
Age (Years)				
Young (0–36)	Baseline			
Middle Age (37–49)	−0.01	0.14	0.99 (0.76,1.29)	0.96
Senior (>50)	0.13	0.14	1.14 (0.86,1.51)	0.36
Ethnicity				
European and Other	Baseline			
Māori and Pacific Peoples	0.33	0.18	1.39 (0.99,1.96)	0.06 *
Unknown	−0.36	0.24	0.70 (0.43,1.12)	0.13
Deprivation Status				
Most Deprived	Baseline			
Least Deprived	0.49	0.18	1.64 (1.15,2.33)	0.01 *
Moderately Deprived	0.26	0.16	1.30 (0.95,1.78)	0.1
Unknown	−0.03	0.18	0.97 (0.69,1.38)	0.88
Serovar				
Ballum	Baseline			
Exotic	1.21	0.79	3.37 (0.71,15.97)	0.13
Copenhageni	1.78	0.65	5.96 (1.68,21.17)	0.01 *
Hardjo	−0.35	0.18	0.71 (0.49,1.01)	0.05 *
Pomona	0.13	0.22	1.14 (0.74,1.74)	0.56
Tarassovi	−0.94	0.27	0.39 (0.23,0.66)	<0.01 *
Unknown	−0.03	0.18	0.97 (0.68,1.39)	0.88
Occupation				
Non-Dairy Farmer	Baseline			
Dairy Farmer	0.36	0.17	1.44 (1.02,2.02)	0.04 *
Meat Worker	−0.15	0.17	0.86 (0.61,1.21)	0.38
Other	0.16	0.19	1.17 (0.82,1.69)	0.39
ROE				
No	Baseline			
Unknown	1.14	0.24	0.87 (0.54,1.39)	0.55
Yes	−0.06	0.19	0.94 (0.64,1.39)	0.76
Geographical Location				
Lower North Island	Baseline			
South Island	−0.63	0.27	0.53 (0.31,0.91)	0.02 *
Upper North Island	−0.23	0.26	0.79 (0.64,1.39)	0.38
Report Year	0.03	0.01	1.03 (1.01,1.05)	0.01 *
Season				
Autumn	Baseline			
Winter	−0.38	0.24	0.68 (0.42,1.10)	0.12
Spring	−0.75	0.24	0.47 (0.29,0.76)	<0.01 *
Summer	0.01	0.25	1.01 (0.63,1.64)	0.96

1444 Observations: Log Likelihood = 929.2, Akaike Information Criterion 1924, Adj. OR = Adjusted Odds Ratios, SE = Standard Error, CI= Confidence Interval, *p* = *p*-value, * = Statistically Significant.
